# Paper mulberry leaves as a potential sterilant: evidence from *Microtus fortis*—a laboratory study

**DOI:** 10.3389/fpls.2023.1092792

**Published:** 2023-06-09

**Authors:** Shuangye Wang, Junzhi Chen, Yunlin Zhao, Meiwen Zhang, Chen Zhang, Jianing He, Lichuan Wei, Zhenggang Xu

**Affiliations:** ^1^ College of Resources and Environment, Hunan Agricultural University, Changsha, Hunan, China; ^2^ Key Laboratory of National Forestry and Grassland Administration on Management of Western Forest Bio-Disaster, College of Forestry, Northwest A & F University, Yangling, Shaanxi, China; ^3^ Dongting Lake Station for Wetland Ecosystem Research, Institute of Subtropical Agriculture, The Chinese Academy of Sciences, Changsha, Hunan, China

**Keywords:** *Broussonetia papyrifera*, *Microtus fortis*, rodenticide, organ coefficient, reproduction, sex hormone

## Abstract

**Introduction:**

The Yangtze vole (*Microtus fortis*) is a small herbivorous rodent that usually causes damage to crops and forests in China. Various measures were used to control their population including chemical rodenticides. However, rodenticides may cause secondary damage to the environment and the ecosystem. Therefore, the development of new rodent sterilants is urgent. Considering that some compounds of paper mulberry leaves have been verified that can inhibit the biosynthesis of sexual hormone, we aimed to explore the antifertility effect of paper mulberry leaves on *M. fortis*.

**Methods:**

In this study, voles were divided into three groups including a male group, a female group, and a breeding group, and paper mulberry leaves were added into basal fodder of voles maintained in laboratory, of which the proportion of leaf weight was 50%. In each group, voles were fed with mixed fodder as treatment (BP) and voles were fed with basal fodder as contrast (CK).

**Results and discussion:**

After feeding for more than 1 month, the results indicated that paper mulberry leaves attracted voles to feed, but inhibited their growth and reproduction. Since the second week, food intakes of BP have been significantly higher than CK (*p*< 0.05). However, weights of voles in male and female groups were 72.283 ± 7.394 g and 49.717 ± 2.278 g in the fifth week, and both were significantly reduced compared with their original weight (*p*< 0.05). Meanwhile, testicular volumes of male voles fed with BP were significantly smaller than CK (former: 318.000 ± 44.654 mm^3^, latter: 459.339 ± 108.755 mm^3^); the testosterone level, sperm number, and vitality of BP were obviously weaker than CK. Female uteruses and oophoron of BP grew slower, and the organ coefficients of uterus and oophoron fed BP were both significantly lower than CK (*p*< 0.05). The first reproduction of BP couple voles spent 45 days, while CK spent only 21 days. These results suggest that paper mulberry leaves could be the potential resource to produce sterilants to control rodent populations by delaying their sexual growth and reproduction. If it was practical, the apparent advantages of paper mulberry are that it is an abundant resource and the inhibitory effect could be effective in both male and female individuals. Our conclusion also supports the transformation of rodent management from lethal management to fertility control, which would be more ecologically friendly to agriculture and the ecosystem.

## 1 Introduction

Rodents are the most species-rich mammalian order and have a cosmopolitan distribution with range extensions that are often associated with humans. They comprise approximately 42% of all living mammals and have 2,277 defined species (Romanenko et al., 2012), including various kinds of mice, rats, and voles. Among vertebrates, rodents are primarily responsible for damage to forest and agriculture (Islam et al., 1993; Sterner et al., 1996; White et al., 1998; Mwanjabe et al., 2002; Singleton et al., 2005b; Mulungu et al., 2007; Guo et al., 2013b). Extensive time and effort have been spent in controlling rodents using various physical and chemical methods (Oogjes, 1997; Meerburg et al., 2004). In recent years, chemical methods have been the mainstay of the rodent pest control profession (Zhou et al., 2013; Chen et al., 2020), although there are some potential harmful effects on predators and the environment (Zhang, 2000). Rodenticides are known for their quick action and high toxicity to all rodents. They must also be very palatable and have no antidotes to ensure that a lethal dose is consumed in one feeding. However, rodents have evolved physiological, morphological, and behavioral adaptations to their environment (Pianalto and Yool, 2017), which enable them to resist these chemical rodenticides over time, thus reducing their long-term efficacy in controlling rodent pests. Thus, toxicities of rodenticides were enhanced; for example, the second-generation anticoagulant rodenticides have higher acute toxicities than the first-generation anticoagulant rodenticides (Thomas et al., 2011). The use of anticoagulant rodenticides to kill rodents could harm many non-target organisms including their natural enemies (Nakagawa et al., 2015; Memmott et al., 2017; Wen et al., 2020). Retention time can vary dramatically between rodenticides, but is generally highest in second-generation anticoagulant rodenticides. These chemical rodenticides are usually lethal to the organs of animals. Scholars found that the liver retention time of second-generation anticoagulant rodenticides was much longer than that of first-generation anticoagulant rodenticides (Erickson and Urban, 2002). This long duration of anticoagulant persistence in liver tissues allows bioaccumulation and biomagnification in predatory species (Martínez-Padilla et al., 2017). In general, strict carnivores are unlikely to eat poisoned bait; however, records demonstrated that there were secondary poisoning of wildlife (Laakso et al., 2010). The study found that large carnivores such as contaminated mountain lions (*Puma concolor*) were even killed by the anticoagulant (Riley et al., 2007). Therefore, new rodenticides should consider the danger they pose for non-target organisms.

Scholars proposed making rodents sexually sterile to control rodent damage (Shuster et al., 2018; Jacoblinnert et al., 2022). Plant-based sterilants are rodent control agents that are produced from plants and their extracts. Some studies started to explore plant-based sterilants and had initial success in the 1980s. Since then, various plants and their extracts have been used in rodent fertility control (Rao et al., 1997; Akbarsha and Murugaian, 2000; Mishra and Singh, 2009). As the main food resource for herbivores and omnivores in the wild, plants have evolved defense strategies against animals (Yuan et al., 2009). Studies verified that many plants have antifertility activities (Daniyal and Akram, 2015; Nath and Deb, 2015) and can inhibit ovulation (Morovati et al., 2008), decrease fertility (Kapoor et al., 1974), disturb the estrous cycle (Khanna and Chauhury, 1968), arrest spermatogenesis, reduce testosterone (Pal, 2008), reduce sperm number and motility, and reduce the frequency of impregnation (Mali et al., 2002) in animals. Therefore, compared with hypertoxic rodenticides, plant-based sterilants can maintain the feeding preference of rodents, reduce poisoning (Whisson and Salmon, 2002; Yang et al., 2022), and sustainably control the population in a less extreme manner. However, the harmful effects of these plant substances are also considered for non-target beneficial organisms. In experience, ingestion by livestock often results in fatal poisonings, which caused significant problems to commercial farmers in many countries (Nwude et al., 1977; Tokarnia et al., 1990; Medeiros et al., 2002). This reminds us to pay attention to toxicities of plants to other animals when they will be used to control the specific species. Animals have strong sensory capabilities and memories, which allow for selective foraging of less toxic foods (Freeland and Janzen, 1974). If consuming a toxic diet is necessary, animals will often reduce intake or the number of feeding bouts (Sorensen et al., 2005); their feeding strategies, to a great extent, decide their habitat selection. Interestingly, animals have more remarkable tolerance to toxicities of plants that are found in their habitat than those that grow elsewhere (Twigg et al., 2003). It suggests taking advantage of the local plant materials to control the rodent pests, which may be less harmful to other organisms.

*Broussonetia papyrifera*, known as paper mulberry, provides the raw material used in paper manufacturing (Ryu et al., 2010; Guo et al., 2013a). It is a deciduous tree belonging to the Moraceae family, which is fast-growing and has highly adaptive capacity to diversified environment, and even germinate in drought regions or polluted regions (Yalley et al., 2020). Paper mulberry grows naturally and, as an economic crop, is widely planted in Asia and other Pacific countries (Xi et al., 2013; Han et al., 2016). It has also been used as livestock feed for a long time (Zhang et al., 2009). Several compounds isolated from paper mulberry have positive (e.g., anti-inflammatory and antioxidant) effects on organisms (Ko et al., 2008; Lin et al., 2008). However, Yang et al. (2014) extracted and isolated compounds in *B. papyrifera* leaves, and according to their conclusion, the luteolin, poliothyrsoside, and three new phenolic compounds (broussoside C, broussoside D, and broussoside F) could potentially inhibit estrogen biosynthesis in human ovarian granulosa. The inhibitory effects of paper mulberry leaves on animals are unknown. Estrogens are key hormones regulating the development and function of reproductive organs in all vertebrates (Barakat et al., 2016), and their reproduction may be delayed by inhibiting estrogens.

The Yangtze vole (*Microtus fortis*) is a small herbivorous rodent widely distributed around wetlands of China (Zhang et al., 2013). Because of the cultivation of lake beaches, soil erosion, and the establishment of a hydropower station in the upper reaches of the Yangtze River Valley, the *M. fortis* population has exploded since the 1970s (Zhang et al., 2014). When water levels rise during the flood season, voles are forced to migrate into the surrounding areas, where they cause serious damage to crops and forests (Xu et al., 2015). Researchers have explored methods to prevent *M. fortis* population increase and their consequent damage, including building rodent-proof walls (Zhang et al., 2012), artificial killing, and applying chemical rodenticides (Zeng and Hu, 2006). However, environmentally neutral and sustainable methods, such as plant-based sterilants, are also needed. Compared with conventional methods, plant-based sterilants were more environmental and economic. In addition, paper mulberry leaves were the available materials that were expediently obtained around regions with rodent pests. The great palatability of paper mulberry leaves for animals has been known, which may imply that the leaves can be substitute food for rodents in croplands, to prevent them from destroying crops. On the other hand, verifying the antifertility activity of paper mulberry leaves on rodents is also important for exploring its utilization. Combined with its palatability, if the antifertility activity was certified on rodents, the advantage of paper mulberry leaves is significant to produce rodent sterilants. Thus, this study explored the effect of paper mulberry leaves as food for *M. fortis*. We assessed their feeding preferences and variations in reproduction, which could provide a scientific reference for developing plant-based sterilants.

## 2 Materials and methods

### 2.1 Fodder preparation

Fresh leaves were collected from paper mulberry trees in Xiangtang City, China (E112°45′–112°55′, N27°53′–28°03′), which were ground into powder after being dried. Moreover, the basal fodder was provided by Hunan SJA Laboratory Animal Co., Ltd. and contained crude protein and crude fat with a calorific value of 20%, 4.8%, or 17.1% (Zhu et al., 2011). The basal fodder was also ground into powder. Thus, two types of fodder were prepared: the powdered basal fodder (CK) and the treated fodder that mixed paper mulberry leaves in basic fodder, in which the weight proportion of leaves was 50% (BP).

### 2.2 Experiment design

The *M. fortis* that were used in the present study were the offspring of wild-caught individuals, which were captured from the Dongting Lake area and maintained in the laboratory, and outbred stock. *M. fortis* is an important pest in agriculture and forestry (Xu et al., 2015). The feeding ability of the different sexes of *M. fortis* was evaluated, including a male group, a female group, and a breeding group. There were two individuals in each cage: the male and female groups had same-sex cages while one male and one female were in each cage for the breeding group. The BP fodder was considered as the experimental treatment and the CK fodder was considered as the contrastive treatment. In total, 36 60-day-old voles were fed and maintained in organic glass cages. There were dismountable troughs in the cages and the rearing temperature was controlled at 20–22°C with 12-h light/dark cycles. Food and water were plentiful throughout the experiments.

The initial body weights of the voles were measured before the experiments and the mean weight in each group was calculated. Then, each individual was measured weekly for 5 weeks. The original and final weights of the voles were compared to evaluate the differences between the CK- and BP-fed voles in each group.

At the same time, the consumption of fodder and foraging behaviors were inspected. The daily consumption of fodder was regarded as food intake per body weight and was used for analysis in this study. Weekly videos were also recorded for 5 weeks, the first of which was carried out after feeding for 3 days. The cages were recorded for 1 h (one by one over 2 days) and the next videos of each cage were recorded after an interval of 7 days. Using the videos, the foraging numbers and foraging duration times were counted.

Until day 10, the reproduction situation in each cage for the breeding group was inspected every day. When fetal voles appeared, we recorded the number of fetuses, then removed the fetuses using clean tweezers and weighed them all together. Moreover, the time of the reproduction event was calculated either from the beginning of the experiment or from the previous reproduction event.

The animal study protocol was approved by the Ethics Committee of Institute of Subtropical Agriculture, The Chinese Academy of Sciences with protocol code: U20A20118 approved on 1 December 2021.

### 2.3 Anatomy and morphological index determination

The voles in the male group and female group were euthanized to collect the organs after being fed for more than 6 weeks. Each mouse was narcotized using diethyl ether (Han et al., 2015) and weighed, then the hearts, livers, lungs, kidneys, male testes, female uteruses, and oophoron were removed. All organs were weighed; furthermore, the lengths, widths, and heights of the male testes were also measured. The organ coefficients were calculated using the weights of the organs divided by the weights of the bodies, then the coefficients of the hearts, livers, lungs, kidneys, male testes, female uteruses, and oophoron of each individual were calculated. Testicular volumes were estimated using the equation for the volume of a prolate spheroid (McCoard et al., 2001; Hermann et al., 2007)


V=4π /3(A ×B×C)

In the equation, A, B, and C represent the long radius, middle radius, and short radius of testes, respectively.

### 2.4 Hormone determination

Blood samples were collected immediately after the voles were narcotized by removing the eyeballs (Deng et al., 2018). The blood samples were kept at room temperature for 1 h. Then, the blood samples were centrifuged twice at 5,000 rpm for 10 min and the serum was transferred into new tubes. The chemiluminescent immunoassay method (Yang et al., 2020) was used to determine the quantity of the male testosterone and female estradiol. The mean values of male testosterone and female estradiol were calculated for the CK- and BP-fed voles.

### 2.5 Sperm observations

Sperm samples were collected from the epididymides after putting in normal saline at 37°C and then counted using a blood counting chamber (Yang et al., 2012). The quantity of sperms in the corner and central counters were recorded using a microscope and the sum total for each individual was calculated. In addition, at least five slides for each individual were selected randomly and magnified 40 times under the microscope and then the numbers of active and static sperms were recorded over 20 min. According to the ratio of active sperms in each slide, the mean values for the CK- and BP-fed voles were calculated.

### 2.6 Data statistics

The mean values were calculated for the CK- and BP-fed voles in each group using SPSS software, and expressed as mean ± SD. The chi-square test was used to compare the variations in weights between the CK- and BP-fed voles. The Mann–Whitney *U* test was used to estimate the differences in foraging numbers, and the independent samples *t*-test was used to evaluate the differences in fodder consumptions, foraging durations, organ coefficients, the contents of the sex hormones, and the characteristics of the sperms between the CK- and BP-fed voles.

## 3 Results and analysis

### 3.1 Consumption of fodder and foraging numbers

**Figure 1:**
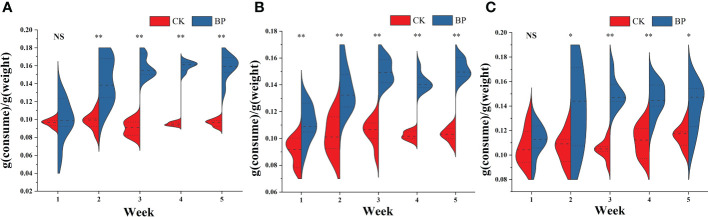
The fodder consumption of *M. fortis* per unit weight: **(A)** male group; **(B)** female group; **(C)** breeding group. NS represents *p* > 0.05; * represents 0.01< *p*< 0.05; ** represents *p*< 0.01. CK was the basic fodder of mice in laboratory, and BP was the mixed fodder constituted by 50% weight of paper mulberry leaves and 50% weight of basic fodder.

The consumptions of fodder were analyzed over 5 weeks for each group (**Figure 1**). For the CK-fed voles, the food intakes were relatively stable (male group: 0.095 ± 0.006 g/g; female group: 0.101 ± 0.010 g/g; breeding group: 0.109 ± 0.011g/g), while the food intakes of the BP-fed voles changed as the experiment proceeded. In the first week, the consumption of the BP feed in the female group ranged from 0.900 g/g to 0.126 g/g, which was significantly higher than that of the CK feed that ranged from 0.777 g/g to 0.1003 g/g (*p*< 0.01). There was no significant difference in consumption rates between the BP and CK feed in the male group or the breeding group (*p* > 0.05). Then, in the second week, the consumption of the BP feed was significantly more than that of the CK feed in all groups (*p*< 0.05). In addition, the range of fodder consumptions reduced gradually after the second week, especially in male and female groups.

**Figure 2:**
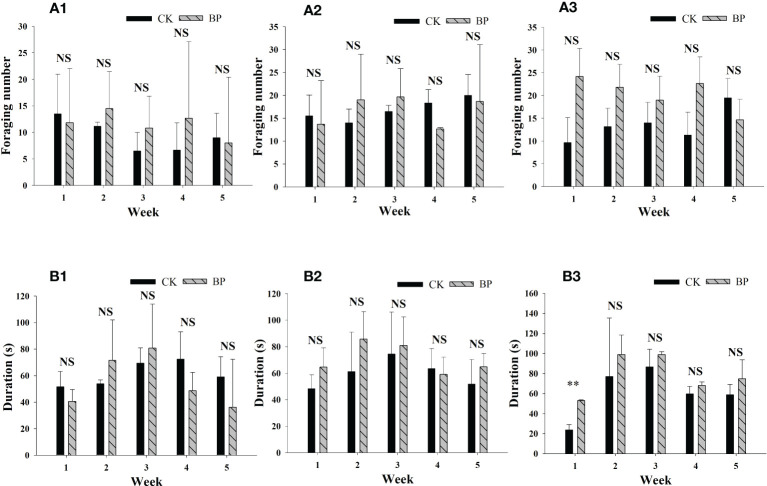
The foraging characteristics in the different groups of *M. fortis*: **(A)** foraging number; **(B)** foraging duration; (1) male group; (2) female group; (3) breeding group. NS represents *p* > 0.05; ** represents *p*< 0.01.

The foraging numbers and duration times were also recorded for 5 weeks (**Figure 2**). The foraging numbers and the feeding times of the different voles varied greatly. Foraging numbers ranged from 6.500 ± 3.500 to 13.500 ± 7.500 and 14.000 ± 3.042 per hour for CK-fed voles in the male group and female group, respectively, and from 8.000 ± 13.423 to 14.500 ± 6.928 and from 12.667 ± 0.289 to 19.667 ± 6.171 per hour for BP-fed voles, respectively. There was no significant difference in the foraging numbers between the CK- and BP-fed voles in the male group or female group (*p* > 0.05). In the breeding group, the foraging numbers of the BP-fed voles were higher than the foraging numbers of the CK-fed voles, though the difference was not significant (*p* > 0.05). The foraging duration times increased from the first week to the second week in all three groups and reached higher levels in the second and third weeks. Similar to the foraging numbers, there was no significant difference in the foraging duration times between the BP- and CK-fed voles in the female and male groups (*p* > 0.05). The foraging times of the BP-fed voles were also higher than those of the CK-fed voles in the breeding group. The differences were not significant between the CK- and BP-fed voles, except in the first week in breeding group when the foraging duration of the BP-fed voles was 52.942 ± 0.786 s, which was much longer than that of the CK-fed voles (23.819 ± 5.335 s; *p*< 0.01).

### 3.2 Variations in weight in each group

**Figure 3:**
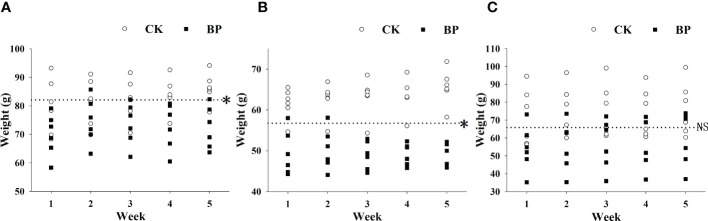
The variations in weight in the different groups of *M. fortis*: **(A)** male group; **(B)** female group; **(C)** breeding group. * represents *p*< 0.05; NS represents *p* > 0.05. The dotted line represents the mean original weight.

The BP feed had a significant effect on the body weights of female and male voles (*p*< 0.05). The weights of BP-fed individuals were significantly lower than those of CK-fed voles in both the male and female groups (**Figures 3A, B**). The original mean weights of the male group and female group were 82.075 ± 13.173 g and 56.733 ± 8.116 g, respectively, but the mean weights of the BP-fed voles in the fifth week were 72.283 ± 7.394 g and 49.717 ± 2.278 g, respectively. Interestingly, there was no significant difference in the variations in weight between CK- and BP-fed individuals in the breeding group (*p* > 0.05; **Figure 3C**).

### 3.3 Effects on the organ coefficients of *M. fortis*

**Figure 4:**
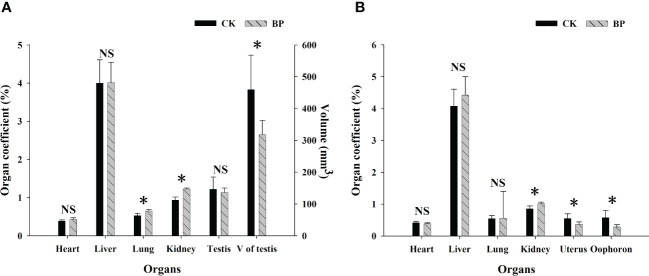
The organ coefficients in the male and female groups: **(A)** male group; **(B)** female group. * represents *p*< 0.05; NS represents *p* > 0.05.

The effects of paper mulberry leaves on the reproductive organs of *M. fortis* were different from those on other organs (**Figure 4**). The BP feed showed a negative influence on reproductive organs in the male and female groups, in which the organ coefficients of the uteruses and oophoron were significantly lower in BP-fed voles than those in CK-fed voles (*p*< 0.05). Although there was no significant difference in the organ coefficients of testes between BP- and CK-fed voles (*p* > 0.05), the mean testicular volume of CK-fed voles was 459.339 ± 108.755 mm^3^, which was significantly larger than that of BP-fed voles (318.000 ± 44.654 mm^3^; *p*< 0.05). The organ coefficients of the hearts and livers did not show any significant difference between males and females (*p* > 0.05), but a higher organ coefficient of the kidneys was observed in the BP-fed voles (*p*< 0.05). The organ coefficient of the lungs was higher in BP-fed voles than CK-fed voles in the male group (*p*< 0.05), while there was no significant difference between the CK- and BP-fed voles in the female group (*p* > 0.05).

### 3.4 Characteristics of the sex hormones and sperm

**Figure 5:**
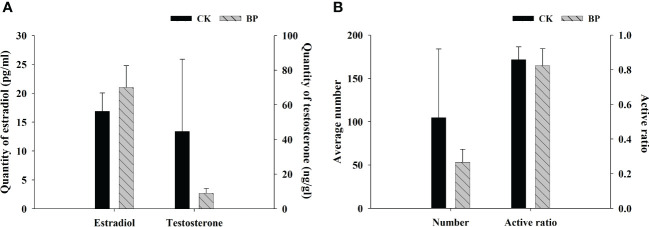
The contents of the sex hormones **(A)** and the characteristic of the sperm **(B)**.

The content of testosterone in CK-fed male individuals was 44.618 ± 46.635 ng/g, which was much higher than that of BP-fed male individuals (8.960 ± 3.395 ng/g; **Figure 5A**). The number and ratio of active sperm in CK-fed males were 104.800 ± 88.525 and 85.82% ± 8.56%, respectively, both of which were higher than those of BP-fed males (53.330 ± 18.148 and 82.39% ± 11.92%, respectively; **Figure 5B**). The variations in the standard deviations of CK-fed individuals were obviously larger than those of BP-fed individuals, especially in terms of testosterone and sperm number. However, the content of estradiol in BP-fed individuals was higher than that of CK-fed individuals (**Figure 5A**).

### 3.5 Breeding characteristics

Reproduction was recorded twice among CK-fed voles, but only once for BP-fed voles (**Table 1**). The first and second reproduction events of CK-fed voles lasted 21 days and 22 days, respectively, but the reproduction event of BP-fed voles lasted 45 days, which was much more delayed than that of the CK-fed voles. For each reproduction event of the CK-fed voles, the mean number of newborns was 4.5; however, there were only two newborns from the BP-fed voles. Based on the lower number of newborns from the BP-fed voles, the mean weight of the newborns was 4.700 g, which was a little heavier than that from the CK-fed voles (3.878 g and 4.524 g) for the two reproduction events.

**Table 1:** The reproductive characteristics in the breeding group.

Fodder	Frequency of Reproduction	Mean Number of Newborns	Mean Weight of Newborns (g)	Mean Reproduction Time (Days)
CK	1	4.5	3.878	21
	2	4.5	4.524	22
BP	1	2	4.700	45

## 4 Discussion

Farmers in many parts of the world, particularly those in developing countries, tend to view economic losses due to rodents as unavoidable (Posamentier, 1997). With recent major changes in the management of cropping systems, whereby farmers do not plow paddocks or burn stubbles frequently and plant seeds by directly drilling, there is increased food and shelter available for rodents (Singleton et al., 2005a) in agricultural lands. Various methods have been used to avoid damage by rodent pests (Zhou et al., 2013; Liu et al., 2018), and more studies have gone into population control to manage the damage. However, rodent habits and the diversity of their habitats make rodent populations difficult to control using established methods. Established methods usually restrict the rodents’ activity range based on forecasts of irruptions or intensively kill them when their population breaks out. Neither strategy can control the population environmentally or sustainably. If the populations could be further reduced by decreasing their reproductive output, low densities could be maintained for longer periods, and damage to crops and forests could be minimized. Fertility control, if delivered efficiently and effectively, could theoretically maintain rodent numbers at low levels (Chambers et al., 1999; Davis et al., 2003). Given the feeding freedom of rodents, palatability should be considered in developing sustainable rodenticides or sterilants.

Our study showed that paper mulberry leaves inhibited the fecundity of *M. fortis*. In addition, during the feeding period, *M. fortis* fed more on fodder mixed with paper mulberry leaves than on basal fodder. We also observed the lower organ coefficients of reproductive organs, delayed reproduction, and a reduction in the number of offspring. As a potential resource for rodent control, paper mulberry leaves satisfied the requirement for both palatability and inhibitory effects. Non-lethal fertility control has been suggested as a better alternative to lethal population management techniques, such as traps, the release of disease or predators, and poisoning (Chambers et al., 1999). Current fertility-control tools include immunocontraception and chemosterilization. Immunocontraception uses an individual’s immune system to inhibit fertility (Hardy et al., 2006), whereas chemosterilization uses a chemical or hormonal compound (Alemany et al., 2008). Although immunocontraception can potentially be very effective, it has severe drawbacks. The major drawbacks of this technology are the attenuation of virulence, innate and acquired resistance of rodents, and different transmission rates (McLeod et al., 2007; Arthur et al., 2009). In addition, chemosterilization is expensive and labor-intensive (Churchill, 2014). Compared to these methods, paper mulberry may be an economically viable resource for developing a rodent sterilant. As a naturally occurring species, the leaf resource is abundant and is effective against *M. fortis* through simple feeding. Furthermore, in our experiment, there was negligible damage to non-reproductive organs, which suggests that *M. fortis* could evolve habits to accept or even like this sterilant, and may avoid harming non-target animals. In contrast, current rodenticides, such as anticoagulants, which are widely used worldwide, usually harm the somatic function of rodents (Bender et al., 1988).

Studies have demonstrated that plant extracts can be used as rodenticides (Teshome et al., 2010; Robinson and Sisco, 2019). Although scholars have worked on the chemosterilization of rodents, most have shown effective results for only one sex (Sarkar et al., 2000; Tran and Hinds, 2013). Thus, one significant advantage of paper mulberry is that it inhibits the growth of reproductive organs in both male and female voles.

Two avenues for future research have emerged from this study: one is to extract and isolate the active compounds from paper mulberry that inhibit the fecundity of *M. fortis* and explore the pathway by which this effect occurs. The second is to explore whether the effects of paper mulberry leaves are universal by determining whether these effects can be duplicated in field populations of *M. fortis* and other rodent pest species. Moreover, vole numbers may be increased through use in further experimentation relating to the sterilant properties of paper mulberry leaves. As a dynamic field, new technologies must be more effective in managing rodent populations than established methods. With the move away from lethal management to fertility control, rodent population management will become more socially acceptable, viable, and effective at the agriculture and ecosystem scale.

## 5 Conclusions

This study provides a scientific reference for the development of sterilants in the management of vole populations. Paper mulberry leaves inhibited the fecundity of *M. fortis*. After feeding on paper mulberry leaves, the voles were not poisoned and showed negligible damage in non-reproductive organs but displayed inhibited growth of the reproductive organs. The female uterus coefficients and oophoron coefficients were significantly lower than that fed basal fodder, and male testicular volume was also inferior when voles were fed the leaves. In addition, male reproductive parameters were weaker after feeding paper mulberry leaves, including testosterone level and sperm count. Unlike traditional rodenticides, paper mulberry leaves might be a potentially sustainable resource that controls rodent populations by delaying their reproductive period. It also follows an orientation that manages the rodent populations by controlling fecundity instead of lethal management.

Undeniably, in theory and practice, this study only scratched the surface of natural phenomena, and more work is required in further studies. For example, what substance does paper mulberry produce that works in rodents, and is this substance more effective after being extracted? With further studies, using paper mulberry leaves could become an effective strategy for managing rodent pests in agriculture and forests.

## Data availability statement

The original contributions presented in the study are included in the article/supplementary material. Further inquiries can be directed to the corresponding author.

## Ethics statement

The animal study protocol was approved by the Ethics Committee of Institute of Subtropical Agriculture, The Chinese Academy of Sciences with protocol code: U20A20118 approved on 1th Dec. 2021.

## Author contributions

The experiments were conceived and designed by YZ, MZ, and ZX. The experiments were carried out by SW, JH, and CZ. SW, JC, and LW analyzed the data. SW contributed to manuscript writing. YZ and ZX revised the manuscript. All authors contributed to the article and approved the submitted version.
